# Behavior of hyperreflective foci in non-infectious uveitic macular edema, a 12-month follow-up prospective study

**DOI:** 10.1186/s12886-018-0848-5

**Published:** 2018-07-20

**Authors:** Barbara Berasategui, Alex Fonollosa, Joseba Artaraz, Ioana Ruiz-Arruza, Jose Ríos, Jessica Matas, Victor Llorenç, David Diaz-Valle, Marina Sastre-Ibañez, Pedro Arriola-Villalobos, Alfredo Adan

**Affiliations:** 1 Department of Ophthalmology, BioCruces Health Research Institute, Cruces Hospital, University of the Basque Country, Cruces square s/n, CP 48903 Baracaldo, Vizcaya Spain; 2 Autoimmune Diseases Research Unit, Department of Internal Medicine, BioCruces Health Research Institute, Cruces Hospital, University of the Basque Country, Bilbao, Spain; 3 Medical Statistics Core Facility, Institut d’Investigacions Biomèdiques August Pi i Sunyer (IDIBAPS), and Hospital Clinic, Barcelona, Spain; 4 Biostatistics Unit, Faculty of Medicine, Universitat Autònoma de Barcelona, Barcelona, Spain; 5 Ophthalmology Institute, Hospital Clinic of Barcelona, Barcelona, Spain; 6 Ophthalmology Department and Health Research Institute (IdISSC), Hospital Clinico San Carlos, Madrid, Spain

**Keywords:** Hyperreflective foci, Intraocular inflammation, Microglia, Optical coherence tomography, Uveitic macular edema, Uveitis

## Abstract

**Background:**

Hyperreflective foci have been described in OCT imaging of patients with retinal vascular diseases. It has been suggested that they may play a role as a prognostic factor of visual outcomes in these diseases. The purpose of this study is to describe the presence of hyperreflective foci in patients with non-infectious uveitic macular edema and evaluate their behavior after treatment.

**Methods:**

We conducted a multicenter**,** prospective, observational, 12-month follow-up study. Inclusion criteria were age > 18 years and a diagnosis of non-infectious uveitic macular edema, defined as central macular thickness of > 300 μm as measured by OCT and fluid in the macula. Collected data included best corrected visual acuity, central macular thickness and the presence, number and distribution (inner or outer retinal layers) of hyperreflective foci. Evaluations were performed at baseline, and at 1, 3, 6, and 12 months after starting treatment.

**Results:**

We included 24 eyes of 24 patients. The frequency of patients with ≥11 hyperreflective foci was 58.4% at baseline, falling to 20.8% at 12 months. Further, hyperreflective foci were observed in the outer retinal layers in 50% of patients at baseline and just 28.6% at 12 months. Mean LogMAR visual acuity improved from 0.55 (95% CI 0.4–0.71) at baseline to 0.22 (95% CI 0.08–0.35) at 12 months (*p* < 0.001). Mean central macular thickness decreased from 453.83 μm (95% CI 396.6–511) at baseline to 269.32 μm (95% CI 227.7–310.9) at 12 months (*P* < 0.001). Central macular thickness was associated with number (*p* = 0.017) and distribution (*p* = 0.004) of hyperreflective foci.

**Conclusions:**

We have observed hyperreflective foci in most of our patients with non-infectious uveitic macular edema. During follow-up and after treatment, the number of foci diminished and they tended to be located in the inner layers of the retina.

## Background

Macular edema is the main cause of vision loss in patients with uveitis [1]. Spectral domain optical coherence tomography (SD-OCT) is the gold standard for the diagnosis of this condition. Retinal thickness has come to be recognized as a remarkably valuable measure in the management of patients with uveitic macular edema (UME) and is almost universally used as a main outcome measure in clinical trials evaluating treatments in uveitis. Qualitative data provided by SD-OCT, i.e., the presence of subretinal fluid, distribution of cysts, and ellipsoid zone status, have also been considered in some papers where the analysis of these data has contributed to understanding the pathogenesis and prognosis of UME [2, 3].

In recent years, hyperreflective foci (HRF) have been described in SD-OCT imaging of patients with macular edema secondary to diabetic retinopathy [4], retinal vein occlusions [5], type 2 macular telangiectasia [6], and age-related macular degeneration [7]. Though their origin is not clear, it has been shown that the abundance of such foci may vary after treatment and has been suggested that there may be an association between a decrease in HRF and an improvement in visual function [8, 9]. The aim of this study was to evaluate the presence and behavior of HRF in UME. In addition, we assessed the potential association between these foci on macular thickness and visual acuity (VA).

## Methods

### Population

In this multicenter, prospective, observational, 12-month follow-up study, we included 24 eyes of 24 patients with UME. Patients were recruited from three referral centers for ocular inflammatory diseases in Spain (Hospital Clinic -Barcelona-, Hospital Universitario Cruces -Bilbao- and Hospital Clinico San Carlos –Madrid-) from january 2014 until september 2014. Local Ethics Committees approved the study (Comité ético de Investigación Clínica del Hospital Clínic de Barcelona 2013/8574; Comité de ética de la investigación con medicamentos de Euskadi, Hospital universitario Cruces PI201406; Comité ético de investigación clínica del hospital clínico San Carlos de Madrid 13/244-E). Informed consent was then obtained from each patient and the research was carried out in accordance with the Declaration of Helsinki.

Inclusion criteria were age > 18 years, and a diagnosis of macular edema (defined as central macular thickness [CMT] of > 300 μm as measured by OCT and fluid in the macula) secondary to non-infectious uveitis. Exclusion criteria were a diagnosis of infectious uveitis or any other retinal disease, a history of intraocular surgery in the last 4 months, and low quality OCT imaging that precluded adequate assessments.

Type of treatment for macular edema was left to the discretion of the treating physician.

The Standardization of Uveitis Nomenclature Working Group criteria were used to anatomically classify the uveitis [10].

### Protocol-based assessments and other study procedures

For the purpose of this study, the following mandatory protocol-based assessments were performed and are reported in the present study: at baseline, and at 1, 3, 6 and 12 months after treatment. Other visits at different time-points (i.e., for monitoring pressure or any other reason) were allowed, at the discretion of the treating physician.

During each appointment, all patients underwent a full ophthalmic examination consisting of determination of best corrected visual acuity (BCVA), which was assessed with Snellen charts at a test distance of 6 m, anterior segment biomicroscopy, Goldmann applanation tonometry, 90-D lens biomicroscopy and SD-OCT. Other imaging methods, e.g., fluorescein angiography, were optional and were left to the discretion of the researcher. Inflammatory activity, that is the presence or absence of anterior chamber cells, vitritis or posterior segment inflammatory signs as judged by the investigator, was recorded at each protocol-based visit.

#### SD-OCT

A Cirrus OCT device (version 4.0, Carl Zeiss Meditec, Dublin, CA) was used in all patients. After pupillary dilatation, two scan protocols were performed: the Macular cube 512 × 128 A-scan, within a 6 × 6 mm^2^ area centered on the fovea; and the Enhanced High Definition Single-Line Raster, which collected data along a 6 mm horizontal line consisting in 4096 A-scans, across the center of the fovea. This single line high definition scan was used to manually count the number of HRF, defined as discrete, punctiform hyperreflective white lesions (as hyperreflective as retinal pigment epithelium), and determine their distribution. As in previous publications [8], the abundance of HRF was assessed semi-quantitatively, each case being assigned to one of four groups: group A, 0 foci; group B, 1 to 10; group C, 11 to 20; and group D, more than 20 foci. Regarding the distribution of HRF, two locations were considered: the inner retina (IR), from the nerve fiber layer to the outer plexiform layer; and the outer retina (OR), from the outer nuclear layer to retinal pigment epithelium. When HRF were localized exclusively in the IR, the case was assigned to group 1, and if there were HRF in the OR (with or without foci in the IR) the case was assigned to group 2, while cases with no HRF were assigned to group 0.

All these assessments of the images were performed by two independent, experienced graders (AF and BB, from one of the participating centers) who were blind to clinical data of the corresponding patients. In the event of discrepancies, the two graders made the assessment together and reached a consensus.

### Statistics

BCVA was converted to the logarithm of the minimum angle of resolution (logMAR) equivalents for statistical analyses. Qualitative variables have been described with percentages or frequencies. Results of logMAR VA and CMT are shown as estimated means and their 95% confidence intervals (95% CI). Other quantitative variables have been described using medians and ranges.

The evolution of LogMAR VA and CMT values has been estimated with a longitudinal lineal model using Generalized Estimated Equations methodology (GEE).

Estimations of LogMAR VA have been performed unadjusted (crude estimation) and adjusted for CMT, amount of HRF and distribution of HRF in order to assess a possible influence of these on VA. Estimations of CMT have been performed unadjusted (crude estimation) and adjusted for amount of HRF and distribution of HRF, in order to assess a possible influence of these on CMT.

GEE models use an unstructured matrix of correlations in order to account for intrasubject variability. All statistical analyses were performed using the Statistical Package for the Social Sciences (SPSS version 20.0 for Windows; SPSS Inc., Chicago, IL). *P* < 0.05 was considered statistically significant for all analyses.

## Results

### Baseline characteristics and clinical course

A total of 24 eyes from 24 patients (17 women) were included. The median age of the group was 49 years (21–67). Anatomic diagnosis classified five cases as anterior uveitis, five as intermediate, eight as posterior and six as panuveitis. Table 1 displays patients’ demographic data, causes of uveitis and treatments for macular edema.

**Table 1:** Patients’ demographic data, causes of uveitis and treatments for macular edema

Patient	Gender	Age	SUN	Cause	Treatment
1	Male	34	Anterior	HLA-B27	Periocular Triamcinolone
2	Male	40	Anterior	HLA-B27	Intravitreal dexamethasone
3	Male	48	Anterior	HLA-B27	Oral steroids
4	Male	37	Anterior	Idiopathic	Oral steroids
5	Female	23	Anterior	Idiopathic	Oral steroids
6	Female	28	Intermediate	Idiopathic	Periocular Triamcinolone
7	Female	25	Intermediate	Idiopathic	Periocular Triamcinolone
8	Female	53	Intermediate	Idiopathic	Periocular Triamcinolone
9	Female	50	Intermediate	Idiopathic	Intravitreal dexamethasone
10	Female	35	Intermediate	Idiopathic	Intravitreal dexamethasone
11	Female	63	Posterior	Sarcoidosis	Oral steroids + Methotrexate
12	Female	54	Posterior	Sarcoidosis	Oral steroids + Methotrexate
13	Female	57	Posterior	Sarcoidosis	Oral steroids + Adalimumab
14	Female	61	Posterior	Sarcoidosis	Oral steroids + Adalimumab
15	Male	61	Posterior	Birdshot	Oral steroids + Tocilizumab
16	Male	34	Posterior	Birdshot	Oral steroids + Cyclosporine
17	Female	59	Posterior	Idiopathic	Oral steroids
18	Female	50	Posterior	Idiopathic	Oral steroids
19	Female	67	Panuveitis	Sarcoidosis	Oral steroids + Adalimumab
20	Female	21	Panuveitis	Sarcoidosis	Oral steroids + Adalimumab
21	Female	35	Panuveitis	Chronic VKH	Oral steroids + Azathioprine
22	Female	47	Panuveitis	Chronic VKH	Periocular triamcinolone+Azathioprine
23	Female	61	Panuveitis	Idiopathic	Oral steroids
24	Male	53	Panuveitis	Idiopathic	Oral steroids

**Fig. 1:**
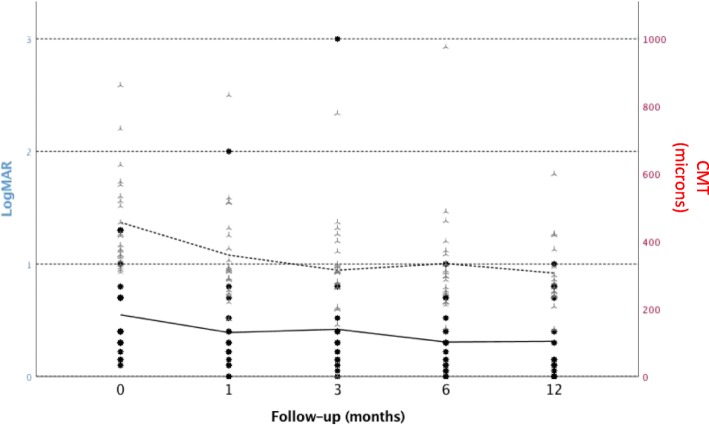
Evolution of logMAR visual acuity (solid line) and central macular thickness (CMT, dashed line) during follow-up

The overall logMAR VA improved from 0.55 (0.4–0.71) at baseline, to 0.42 (0.25–0.59) at 1 month (*p* = 0.046), 0.42 (0.18–0.66) at 3 months (*p* = 0.255), 0.31 (0.19–0.42) at 6 months (*p* = 0.001) and 0.22 (0.08–0.35) at 12 months (*p* < 0.001). In parallel, the CMT decreased, from 453.83 μm (396.6–511) at diagnosis, to 358.34 *μ*m (301.69–415) at 1 month (*p* = 0.006), 315.2 *μ*m (258.4–372.1) at 3 months (*p* < 0.001), 328.87 *μ*m (262.3–395.5) at 6 months (*p* = 0.002) and 269.32 *μ*m (227.7–310.9) after 1 year (*p* < 0.001) Fig. 1 shows the evolution of logMAR VA and CMT during follow-up. Regarding the amount of HRF, we observed a progressive reduction in the percentage of eyes classified as group C or D, that is, with ≥11 HRF, from 58.4% at baseline, to 40.5% at 1 month, 45.8% at 3 months, 22.7% at 6 months and 21.7% at 12 months. Concerning the distribution of these foci at baseline, 50% of patients were classified as group 2 and 50% as group 1 or 0. During follow-up, at all time points, fewer than half of the patients were classified as group 2 (that is, patients with HRF in the outer retina) (month 1, 47.7% in group 2 vs 52.3%in group 0 + 1, month 3: 34.7% vs 65.3%, month 6: 28.6% vs 71.4%, and month 12: 28.6% vs 71.4%). Grader’s assessments matched in 111 of 120 scans (92.5%).

**Fig. 2:**
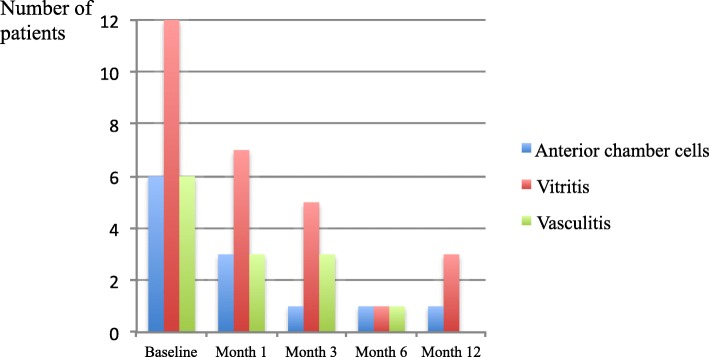
Number of patients with inflammatory activity at each protocol-based visit

Figure 2 shows the number of patients with inflammatory activity at each protocol-based visit.

Table 2 lists logMAR VA and SD-OCT parameters from all patients during follow-up.

**Table 2:** Changes in SD-OCT parameters and visual acuity over follow-up (all patients)

	Baseline	Month 1	Month 3	Month 6	Month 12
Frequency of patients with ≥11 foci(groups C or D)	58.3%	40.5%	45.8%	22.7%	21.7%
Distribution (% patients in each group)					
Group 0 (no foci)	4.2%	4.8%	8.7%	14.3%	4.7%
Group 1 (inner retina)	45.8%	47.5%	56.6%	57.1%	66.7%
Group 2 (outer retina)	50%	47.7%	34.7%	28.6%	28.6%
logMAR VA^a^	0.55 (0.4–0.71)	0.42 (0.25–0.59)	0.42 (0.18–0.66)	0.31 (0.19–0.42)	0.22 (0.08–0.35)
CMT (μm)^b^	453.83 (396.6–511)	358.34 (301.69–415)	315.2 (258.4–372.1)	328.87 (262.3–395.5)	269.32 (227.7–310.9)

^a^logMAR VA: logarithm of the minimum angle of resolution visual acuity

^b^CMT: Central macular thickness

Tables 3, 4, 5 and 6 show logMAR VA and HRF related data in patients with anterior, intermediate, posterior and panuveitis respectively.

**Table 3:** Behavior of foci and evolution of visual acuity in patients with anterior uveitis

	Baseline	Month 1	Month 3	Month 6	Month 12
Frequency of patients with ≥11 foci(groups C or D)	40%	40%	40%	0%	0%
Distribution (% patients in each group)					
Group 0 (no foci)	20%	20%	40%	0%	0%
Group 1 (inner retina)	20%	40%	40%	100%	75%
Group 2 (outer retina)	60%	40%	20%	0%	25%
Mean logMAR VA^a^	0.56	0.32	0.32	0.2	0.125

^a^logMAR VA: logarithm of the minimum angle of resolution visual acuity

**Table 4:** Behavior of foci and evolution of visual acuity in patients with intermediate uveitis

	Baseline	Month 1	Month 3	Month 6	Month 12
Frequency of patients with ≥11 foci(groups C or D)	60%	20%	60%	20%	20%
Distribution (% patients in each group)					
Group 0 (no foci)	0%	0%	0%	0%	25%
Group 1 (inner retina)	40%	100%	80%	100%	50%
Group 2 (outer retina)	60%	0%	20%	0%	25%
Mean logMAR VA^a^	0.28	0.25	0.23	0.24	0.21

^a^ logMAR VA: logarithm of the minimum angle of resolution visual acuity

**Table 5:** Behavior of foci and evolution of visual acuity in patients with posterior uveitis

	Baseline	Month 1	Month 3	Month 6	Month 12
Frequency of patients with ≥11 foci(groups C or D)	62.5%	28.5%	25%	14.2%	12.5%
Distribution (% patients in each group)					
Group 0 (no foci)	0%	0%	0%	0%	0%
Group 1 (inner retina)	50%	28.5%	57%	62.5%	85.7%
Group 2 (outer retina)	50%	71.5%	43%	37.5%	14.3%
Mean logMAR VA^a^	0.5	0.3	0.41	0.25	0.18

^a^ logMAR VA: logarithm of the minimum angle of resolution visual acuity

**Table 6:** Behavior of foci and evolution of visual acuity in patients with panuveitis uveitis

	Baseline	Month 1	Month 3	Month 6	Month 12
Frequency of patients with ≥11 foci(groups C or D)	66.7%	80%	66.7%	60%	60%
Distribution (% patients in each group)					
Group 0 (no foci)	0%	0%	0%	16.7%	0%
Group 1 (inner retina)	67%	40%	50%	33.3%	50%
Group 2 (outer retina)	33%	60%	50%	50%	50%
Mean logMAR VA^a^	0.85	0.8	0.69	0.55	0.40

^a^ logMAR VA: logarithm of the minimum angle of resolution visual acuity

### Influence of OCT parameters on visual acuity

The adjusted model for the estimation of VA showed that the decrease in CMT was associated with the increase in VA (*p* = 0.002). However VA was not associated with either HRF number or distribution (*p* = 0.513 and *p* = 0.324 respectively). On the other hand, the adjusted model for the estimation of CMT showed that both HRF number (*p* = 0.017) and distribution (*p* = 0.004) had an influence on CMT values, that is, the decrease in CMT was associated with a decrease in the number of HRF and the distribution of the foci.

## Discussion

**Fig. 3:**
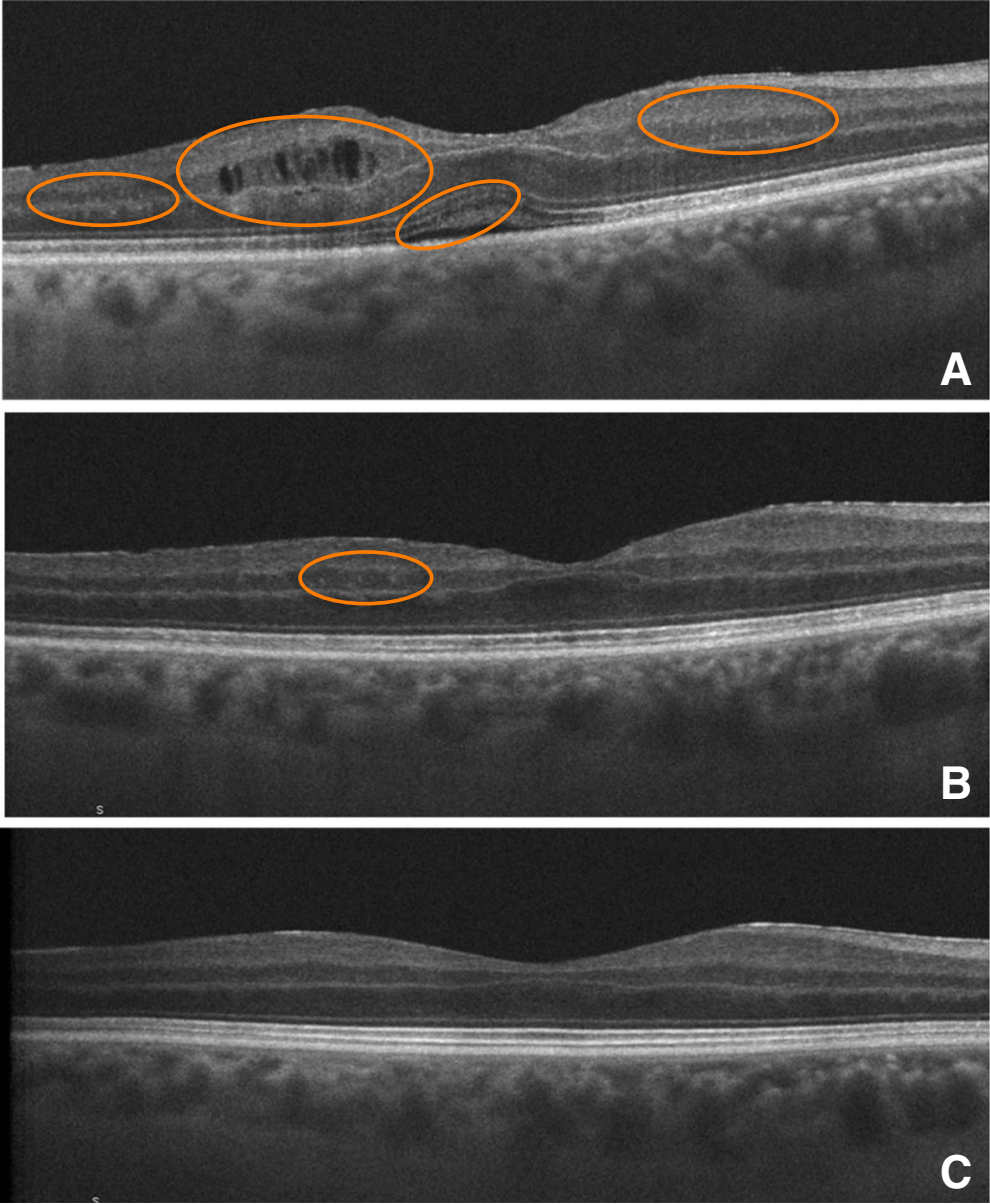
Example of behavior of HRF number and distribution as detected by SD-OCT over the course of follow-up. Circles highlight foci. Left eye of a 61-year-old man with chronic idiopathic anterior uveitis. **a** At baseline, multiple HRF scattered across all retina layers and macular edema (assigned to groups D and 2. **b** At 6 months, HRF number reduced and outer retina not affected (assigned to groups B and 1). **c** At 12 months, no visible foci (assigned to groups A and 0)

In this prospective study we describe, to our knowledge for the first time, the behavior of HRF in patients with UME. At baseline, patients had larger numbers of foci and half of them had at least some foci in the outer retina. During follow-up, while macular edema resolved, OCT showed fewer foci and those that remained were more frequently located in the inner retina. Figure 3 illustrates this behavior. Moreover, macular thickness was found to be associated with both the number and the distribution of the foci.

Although HRF have been described in several diseases including diabetic macular edema, age-related macular degeneration, retinal vein occlusions and type 2 macular telangiectasia, the precise nature of these foci and their molecular constituents remain unclear. Three main theories have been put forward in publications concerning these foci. Some authors have hypothesized that HRF represent precursors of hard exudates [4, 11]. Others suggested that they are degenerated photoreceptors or macrophages engulfing such cells, since they have been observed close to disrupted external limiting membrane and ellipsoid zone and have been associated with decreased VA [12]. Finally, other authors have interpreted them as microglial cells activated during an inflammatory reaction [13, 14]. All these theories are plausible and it is possible that all three mechanisms may occur in the same disease but it is likely that each one of them plays a predominant role in a given disease.

Shape of foci is described as round or oval in previous publications regarding HRF in patients with retinal vascular diseases. Our cases showed also round or oval foci. Regarding the size, hyperreflective foci are defined as “small” in publications that evaluate this feature in patients with retinal vascular diseases, though precise size is not reported. In SD-OCT figures provided in these publications displaying foci, variable sizes may be observed. Though it is a subjective judgement, we believe that in our uveitic patients, foci are usually smaller than those observed in patients with diabetic macular edema or retinal vein occlusions. We speculate that this may be explained by a different origin of foci in different diseases. In retinal vascular diseases bigger foci may correspond to lipid exudation; on the other hand smaller foci seen in our patients may correspond to inflammatory cells. A microglial and leukocytic origin of the HRF would seem the most plausible in the context of UME, given the clear inflammatory origin of this condition, and the absence of hard exudates in UME. This may support the view that HRF should not be considered an initial lipidic extravasation in these cases. In this regard, a study assessing SD-OCT imaging of the vitreous and retina of seven patients with posterior segment inflammatory disease described HRF of a size consistent with that expected for inflammatory cells [15]. Interestingly, data from studies performed in murine experimental autoimmune uveoretinitis assessing correlations of OCT imaging of inflammatory lesions and their histopathologic analysis have shown that HRF may represent cellular infiltration [16].

In our patients, HRF decreased over time after starting treatment, whilst macular thickness decreased and edema resolved. Similar findings have been described by other researchers in patients with retinal vasculopathies and age-related macular degeneration. In diabetic macular edema, Vujosevic et al. [9] assessed the presence of HRF and the effect of treatment with anti-vascular endothelial growth factor on their abundance. They observed that the number of foci decreased after treatment, but did not find a correlation between the number of HRF and retinal thickness. In patients with macular edema secondary to diabetic retinopathy or branch retinal vein occlusions treated with intravitreal implant of dexamethasone or ranibizumab, Chatziralli et al. observed a decrease in HRF in parallel with resolution of the macular edema [17]. Framme et al. described a reduction in the number of HRF in 54% of their patients with neovascular choroidal neovascularization after treatment with ranibizumab [7]. Moreover, this reduction correlated with a decrease in CMT. Abri Aghdam et al. assessed the behavior of HRF in patients with neovascular age related macular degeneration after treatment with intravitreal aflibercept [18]. They observed a decrease in the number of foci within radius of 500 and 1500 μm, as well as a correlation between the CMT and number of foci within a 500-μm radius. To explain this behavior, these researchers suggested that HRF were precursors of lipid exudates and hence a sign of hyperpermeability, which might explain the association found between number of foci and macular thickness.

In patients with UME, the inflammatory process induces the invasion of leukocytes into the retina and the activation of microglia. These undergo significant changes in shape and size, from ramified multidirectional extensions to polarized dendrites and then to larger rounded cells which aggregate [19]. Leukocytes and activated microglial cells produce cytokines that increase vascular and epithelial permeability. When the inflammation resolves, the level of retinal cellular infiltrates decreases. These phenomena may support our finding of an association between HRF number and macular thickness.

As mentioned above, almost half of our cases showed HRF in the outer retina at baseline. During follow-up, as the edema resolved, foci were more frequently located in the inner retina. Similar observations have been described in diabetic macular edema. Vujosevic et al. [9]_,_ in their study assessing the effect of ranibizumab on HRF in diabetic macular edema, reported that the main decrease in foci occurred in the outer nuclear layer when edema resolved. Zheng et al. [19] showed that resting microglia are physiologically located in the inner retinal layers in human eyes. In the same study it was shown that activated microglia migrate towards the outer retinal layers in human eyes with diabetic macular edema and it is suggested that proinflammatory cytokines are responsible for the activation of microglia. Interestingly, in a rat model of experimental autoimmune uveoretinitis, Rao et al. showed that microglia had migrated from the nerve fiber layer and other inner retinal layers to the photoreceptor layer at day 9 after the induction of uveitis [20]. Moreover, Ding X et al. showed that rat microglial cells activated by lipopolysaccharides secreted proinflammatory cytokines (tumour necrosis factor and interleukin beta) which could promote vascular dysfunction and hence permeability [21]. These findings could explain the high frequency of patients with HRF in the outer retina at baseline, when macular edema was present and hence the inflammatory process was active. It has been shown that glucocorticoids inhibit microglial migration [22]. We speculate that the treatment given in our patients (mainly glucocorticoids) may explain the behavior of the HRF after treatment, that is, a more frequent location of foci in the inner retina.

We have not found the number or location of the HRF to have an independent influence on VA. Previous studies have evaluated possible associations between HRF and visual outcomes in diabetic macular edema, retinal vein occlusions and neovascular age-related macular degeneration. In the study by Vujosevic et al., the number of foci was correlated inversely with retinal sensitivity and directly with non-stable fixation, as measured by microperimetry [9]. Uji et al. found an association between the presence of HRF in the outer retinal layers and poor VA in patients with diabetic macular edema [12]. Moreover, both HRF and VA were associated with disruptions in the external limiting membrane and in the junction between the inner and outer segment of the photoreceptors (nowadays known as the ellipsoid zone). In the study by Chatziralli et al. performed in patients with diabetic macular edema and retinal vein occlusions, a higher number of HRF was associated with poorer VA [17]. Kang et al. found that the number of HRF at baseline was inversely associated with final VA in patients with diabetic macular edema treated with intravitreal bevacizumab [23]. The same group reported similar findings in patients with branch retinal vein occlusion [8], neovascular age-related macular degeneration and polypoidal choroidal neovascular vasculopathy [24].

In most of the aforementioned studies, HRF are assumed to be extravasations of lipoproteins and precursors of lipid exudates and the researchers consistently suggest that the underlying pathogenesis of poorer visual outcomes may be related to a toxic effect of lipids on photoreceptors. Regarding UME, it has been shown in a rat model of experimental autoimmune uveoretinitis that activated microglia located at the photoreceptor layer secrete peroxynitrite, which is the most potent biological oxidant known and capable of oxidizing cellular components [20]. Assuming a microglial origin of the foci, one could expect some deleterious effect on photoreceptors and hence on VA, due to the presence of microglia in the outer retina. We failed, however, to demonstrate an association between BCVA and HRF number or distribution. A protective effect of the treatment administered, usually local or systemic steroids, on photoreceptors and/or an insufficient capability of the BCVA test to highlight functional damage may explain this finding.

The main limitation of our study is the subjective assessment and counting of HRF. Nevertheless, agreement between graders of the scans was high. In the future, software able to automatically measure the amount of HRF may help us objectively define the behavior of such foci and clarify their meaning and relevance. Other limitations are the relatively small size of the sample and that the SD-OCT device used lacks software capable of performing consecutive scans in the same retinal section. Strengths of our study are its prospective nature and the long-term follow-up.

## Conclusions

In conclusion, SD-OCT scans showed HRF in eyes with UME in our study. After treatment, the number of foci decreased and their distribution changed, remaining foci locating preferentially in the inner retina, and this was associated with a decrease in macular thickness. Further studies with larger numbers of patients are needed to confirm these results and shed light on their implications for clinical practice.

## Abbreviations

BCVA: Best corrected visual acuity
CI: Confidence interval
CMT: Central macular thickness
GEE: Generalized estimated equations methodology
HRF: Hyperreflective foci
IR: Inner retina
logMAR: Logarithm of the minimum angle of resolution
OR: Outer retina
SD-OCT: Spectral domain Optical coherence tomography
SPSS: Statistical Package for the Social Sciences
UME: Uveitic macular edema
VA: Visual acuity
